# Application of Massively Parallel Sequencing to Genetic Diagnosis in Multiplex Families with Idiopathic Sensorineural Hearing Impairment

**DOI:** 10.1371/journal.pone.0057369

**Published:** 2013-02-22

**Authors:** Chen-Chi Wu, Yin-Hung Lin, Ying-Chang Lu, Pei-Jer Chen, Wei-Shiung Yang, Chuan-Jen Hsu, Pei-Lung Chen

**Affiliations:** 1 Department of Otolaryngology, National Taiwan University Hospital, Taipei, Taiwan; 2 Department of Medical Genetics, National Taiwan University Hospital, Taipei, Taiwan; 3 Graduate Institute of Molecular Medicine, National Taiwan University College of Medicine, Taipei, Taiwan; 4 Graduate Institute of Clinical Medicine, National Taiwan University College of Medicine, Taipei, Taiwan; 5 Department of Internal Medicine, National Taiwan University Hospital, Taipei, Taiwan; 6 Research Center for Developmental Biology and Regenerative Medicine, National Taiwan University, Taipei, Taiwan; 7 Graduate Institute of Medical Genomics and Proteomics, National Taiwan University College of Medicine, Taipei, Taiwan; Innsbruck Medical University, Austria

## Abstract

Despite the clinical utility of genetic diagnosis to address idiopathic sensorineural hearing impairment (SNHI), the current strategy for screening mutations via Sanger sequencing suffers from the limitation that only a limited number of DNA fragments associated with common deafness mutations can be genotyped. Consequently, a definitive genetic diagnosis cannot be achieved in many families with discernible family history. To investigate the diagnostic utility of massively parallel sequencing (MPS), we applied the MPS technique to 12 multiplex families with idiopathic SNHI in which common deafness mutations had previously been ruled out. NimbleGen sequence capture array was designed to target all protein coding sequences (CDSs) and 100 bp of the flanking sequence of 80 common deafness genes. We performed MPS on the Illumina HiSeq2000, and applied BWA, SAMtools, Picard, GATK, Variant Tools, ANNOVAR, and IGV for bioinformatics analyses. Initial data filtering with allele frequencies (<5% in the 1000 Genomes Project and 5400 NHLBI exomes) and PolyPhen2/SIFT scores (>0.95) prioritized 5 indels (insertions/deletions) and 36 missense variants in the 12 multiplex families. After further validation by Sanger sequencing, segregation pattern, and evolutionary conservation of amino acid residues, we identified 4 variants in 4 different genes, which might lead to SNHI in 4 families compatible with autosomal dominant inheritance. These included *GJB2* p.R75Q, *MYO7A* p.T381M, *KCNQ4* p.S680F, and *MYH9* p.E1256K. Among them, *KCNQ4* p.S680F and *MYH9* p.E1256K were novel. In conclusion, MPS allows genetic diagnosis in multiplex families with idiopathic SNHI by detecting mutations in relatively uncommon deafness genes.

## Introduction

Hearing loss is a common sensory disorder. Approximately 1 in 500 newborns are affected with bilateral moderate or more severe sensorineural hearing impairment (SNHI). Before the age of 5, the prevalence increases to 2.7 per 1,000, and during adolescence, to 3.5 per 1,000 [1]. It has been revealed that more than 50% of children with SNHI have attributable genetic factors [2]. As such, genetic testing is a powerful tool for a precise genetic diagnosis in hearing-impaired children. In addition, this diagnostic approach provides clues concerning the pathogenesis of hearing impairment, and allows for an accurate prognosis [3], [4], facilitates genetic counseling [5], prevents further hearing loss [6], and might help to design novel strategies for future treatment.

However, the genetic heterogeneity of hereditary hearing impairment remains a major obstacle to obtaining molecular diagnoses in the majority of cases. To date, more than 100 genes are associated with deafness, and approximately 50 genes have been identified to cause nonsyndromic hereditary hearing impairment (The Hereditary Hearing Loss Homepage, http://hereditaryhearingloss.org/) [7]. For example, in Taiwan, although mutations in certain genes such as *GJB2* (or *Cx26*) (Gene ID: 2706), *SLC26A4* (or *PDS*) (Gene ID: 5172), and the mitochondrial 12SrRNA gene (*MTRNR1*) (Gene ID: 4549) have been shown to be much more prevalent than other genes, a definitive diagnosis by direct sequencing of these coding regions could only be established in approximately 1/3 patients with idiopathic SNHI [8] and 1/4 patients with cochlear implantation [4], respectively. It is likely that there are other, relatively uncommon, genetic mutations that contribute to SNHI in some of the remaining families, especially those with many affected members (i.e., multiplex families). Recently, the development of massively parallel sequencing (MPS), also known as next-generation sequencing (NGS), which generates millions of DNA sequence reads in parallel during a single experimental run, offers a potential solution to approach hereditary disorders with genetic heterogeneity such as retinitis pigmentosa [9], breast cancer [10], cardiomyopathy [11], and hereditary hearing impairment [12], [13]. To investigate the diagnostic utility of MPS, we applied the MPS technique to a cohort composed of 12 families with idiopathic SNHI in which common deafness mutations had been ruled out as the cause.

## Materials and Methods

### Family Recruitment and Phenotype Characterization

Twelve multiplex families with idiopathic nonsyndromic SNHI, including 10 families compatible with autosomal dominant inheritance and 2 families compatible with autosomal recessive inheritance (Figure S1), were enrolled in the present study. All 12 families were Han Chinese from Taiwan in ethnicity. Prior mutation screening using the SNaPshot multiplex assays, which targeted 20 common deafness-associated mutations in the Han Chinese population of Taiwan [14], did not detect mutations in these 12 families. For the proband and affected family members, comprehensive family history and past medical records were obtained, and physical examination, neurological examination, audiological results, and temporal bone imaging results were obtained and analyzed. Audiological results were assessed and characterized in terms of 2 parameters: hearing levels and audiogram shapes [15]. The hearing level of the better ear, calculated by a 4-tone average (0.5, 1, 2, and 4 kHz), was labeled as mild (20∼40 dBHL), moderate (41∼70 dBHL), severe (71∼95 dBHL), or profound (>95 dBHL) hearing loss (GENDEAF: http://audiology.unife.it/www.gendeaf.org/index.html). Temporal bone imaging results were obtained using high-resolution computed tomography and/or magnetic resonance imaging, and inner ear malformations (IEMs) were identified and determined according to the criteria in the literature [15]. Written informed consent for participation in the project was obtained from the subjects, and all procedures were approved by the Research Ethics Committee of the National Taiwan University Hospital.

### Target Enrichment, Massively Parallel Sequencing and Variant Calling

**Figure 1:**
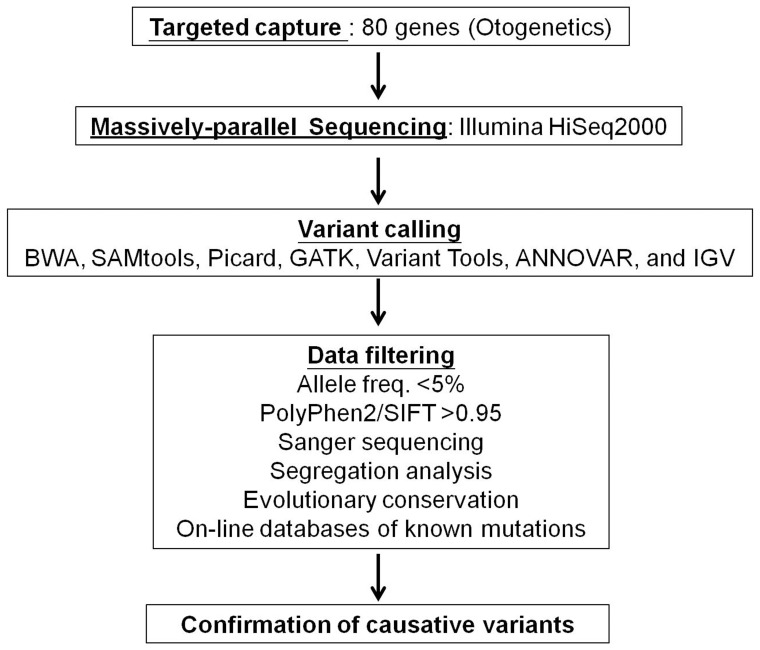
Pipeline to identify causative variants in the present study. Causative variants in the 12 families were confirmed through the steps of targeted capture of 80 known deafness genes; massively parallel sequencing with the Illumina platform; variant calling using the BWA, SAMtools, Picard, GATK, and IGV software packages; and data filtering with various criteria, including allele frequencies <5% in both the 1000 Genomes Project and NHLBI-ESP 5400 exome project, both PolyPhen2 and SIFT scores >0.95, Sanger sequencing, segregation pattern, evolutionary conservation of amino acid residues, and on-line databases of known deafness mutations.

Figure 1 depicts the major criteria for identification of causative variants in the affected families, including target enrichment, massively parallel sequencing, variant calling, and data filtering. Genomic DNA (gDNA) was extracted from the probands of the 12 families and submitted to Otogenetics Corporation (Norcross, GA, USA) for exome capture and sequencing. Briefly, gDNA was subjected to agarose gel and O.D. ratio tests to confirm its purity and concentration prior to Covaris fragmentation (Covaris, Inc., Woburn, MA, USA). Fragmented gDNAs were tested for size distribution and concentration using the Agilent Bioanalyzer 2100 (Agilent Technologies, Inc., Santa Clara, CA, USA) and NanoDrop (Thermo Fisher Scientific, Inc., Wilmington, DE, USA). Illumina libraries were generated from fragmented gDNA (only after the gDNA passed the quality control checks) using NEBNext reagents (New England Biolabs, Ipswich, MA, USA; catalog# E6040), and the resulting libraries were subjected to target enrichment using custom probes targeting the Human Deafness Gene DA1 panel (Otogenetics Corporation) following the manufacturer’s instructions. The libraries were first tested for enrichment by qPCR and for size distribution and concentration using the Agilent Bioanalyzer 2100. The samples were then sequenced on an Illumina HiSeq2000 that generated paired-end reads of 100 nucleotides.

We aligned the raw sequencing data to the latest reference human genome (Feb. 2009, GRCh37/hg19) using the BWA software (version 0.5.9) [16]. We used SAMtools (version 0.1.18) [17] and Picard (version 1.54) (http://picard.sourceforge.net) to perform the necessary data conversion, sorting, and indexing. The main variant calling process, for both indels (insertion/deletions) and single nucleotide variants, was operated by using the GATK software package (version 1.2-59-gd74367c1) [18], [19]. We applied Variant Tools (version 1.0.2) to select and analyze the variants [20]. ANNOVAR (version 2012-03-08) was used to appropriately annotate the genetic variants [21]. This included gene annotation, amino acid change annotation, SIFT scores [22], PolyPhen2 scores [23], dbSNP identifiers, 1000 Genomes Project allele frequencies, and NHLBI-ESP 5400 exome project allele frequencies. We then used IGV (version 2.1.16) to view the mapping and annotation of sequences on a graphic interface [24], [25].

### Data Filtering

After variant calling, we used ANNOVAR to annotate genetic variants, identify variants that are reported in dbSNP135, and check if variants have already been reported in the 1000 Genomes Project (Feb. 2012) or NHLBI-ESP 5400 exome project (all ethnicity). Only variants with allele frequencies <5% in both the 1000 Genomes Project and NHLBI-ESP 5400 exome project were selected. Among these variants, all frameshift and non-frameshift indel variants, nonsense variants, and splice site variants were included for further analysis. In contrast, for missense variants, PolyPhen2 and SIFT (http://sift.jcvi.org/) were used to assess the effect on the protein. SIFT prediction is based on the degree of conservation of amino acid residues in multiple alignment information. The amino acid substitution is predicted damaging if the score is >0.95, and tolerated if the score is <0.95. Accordingly, missense variants with a score <0.95 in PolyPhen2 or SIFT were excluded. Variants meeting the aforementioned criteria were then subjected to Sanger sequencing to confirm the nucleotide change; segregation pattern were obtained to determine if the variant co-segregated with the hearing impairment phenotype in the pedigrees, and the evolutionary conservation of amino acid residues was assessed. Known variants present in databases (http://deafnessvariationdatabase.org/) and published in the literature as disease-causing were regarded as pathogenic mutations. The mutated gene identified in each family was classified as autosomal dominant or autosomal recessive according to the Hereditary Hearing Loss Homepage (http://hereditaryhearingloss.org), and was then correlated to the inheritance pattern in each family. Allele frequencies of the variants detected were also verified in a panel of 100 controls with normal hearing of the same ethnicity.

## Results

### Target Enrichment and Massively Parallel Sequencing

DNA libraries were created from the probands of the 12 multiplex families with SNHI (Table 1). As shown in Supplementary Material, Table S1, the Human Deafness DA1 Panel was designed to enrich for all coding sequences (CDSs) and splicing sites of 80 confirmed human deafness genes. This covered 1,253 exons with a total coding region of 193,634 bp. The enriched targets then were submitted to MPS using the Illumina HiSeq2000 and the paired-end protocol with a length of 100 bp. The average coverage was 490 folds. 99.3% of sequences had coverage greater than 30 folds, 99% greater than 50 folds, and 95.3% greater than 100 folds.

**Table 1:** Number of Variants Detected in the Probands of the 12 Families.

		Variants (freq. <5%)			Potentially functional variants				
Proband	Inheritance	SNP	Private	Total	Nonsense	Missense	Splice	Frameshift	Total
			indels				junctions		
DE2087	ADNSHL	296	4	300	1	3	1	0	5
DE2180	ADNSHL	284	4	288	2	5	1	0	8
DE2548	ADNSHL	298	4	302	1	4	1	0	6
DE2616	ADNSHL	278	4	282	1	3	0	1	5
DE2624	ADNSHL	98	3	101	1	2	1	0	4
DE2675	ADNSHL	138	4	142	1	7	2	0	10
DE2721	ADNSHL	182	4	186	2	10	0	0	12
DE2737	ARNSHL	284	4	288	2	3	0	0	5
DE2827	ADNSHL	109	3	112	1	4	0	0	5
DE2939	ARNSHL	367	8	375	2	0	1	0	3
DE3050	ADNSHL	171	3	174	1	10	0	1	12
DE3281	ADNSHL	148	2	150	1	6	0	0	7

SNP, single nucleotide polymorphism; ADNSHL, autosomal dominant nonsyndromic hearing loss; ARNSHL, autosomal recessive nonsyndromic hearing loss.

### Data Filtering and Identification of Causative Variants

Initial data filtering with allele frequencies and PolyPhen2/SIFT scores prioritized 5 indels and 36 missense variants in the 12 multiplex families. After further validation by Sanger sequencing, segregation pattern, and assessment of evolutionary conservation of amino acid residues, we identified 5 variants in 5 different genes, which might have led to SNHI in 6 of the families compatible with autosomal dominant inheritance. These included *GJB2* p.R75Q, *MYO7A* (GeneID 4647) p.T381M, *KCNQ4* (GeneID 9132) p.S680F, *MYH9* (GeneID 4627) p.E1256K, and *GJB4* (GeneID 127534) p.C169W (Table 2).

**Table 2:** The Variants Identified in the 6 Families Compatible with Autosomal Dominant Inheritance.

Proband	Inheritance	Genomic	Reference	Variant	Total	Gene	cDNA	Protein	SIFT	PolyPhen2	5400NHLBI	1000
		Coordinates	reads	reads	reads		(RefSeq ID)	(RefSeq ID)			exomes	Genomes
DE2624	ADNSHL	Chr13∶20763497	130	120	251	*GJB2*	c.224G>A	p.R75Q	1	1	0	0
		C>T					(NM_004004.5)	(NP_003995.2)				
DE2675	ADNSHL	Chr1∶41304146	156	97	253	*KCNQ4*	c.1877C>T	p.S680F	0.98	0.999	0	0
		C>T					(NM_004700.3)	(NP_004691.2)				
DE2721	ADNSHL	Chr22∶36690209	122	78	201	*MYH9*	c.3766G>A	p.E1256K	1	1	0	0
		C>T					(NM_002473.4)	(NP_002464.1)				
DE2827	ADNSHL	Chr1∶35227362	120	102	222	*GJB4*	c.507C>G	p.C169W	1	1	0.028	0.03
		C>G					(NM_153212.2)	(NP_694944.1)				
DE3050	ADNSHL	Chr11∶76871270	32	41	73	*MYO7A*	c.1142C>T	p.T381M	0.98	0.996	0	0
		C>T					(NM_000260.3)	(NP_000251.3)				
DE3281	ADNSHL	Chr1∶35227362	155	150	305	*GJB4*	c.507C>G	p.C169W	1	1	0.028	0.03
		C>G					(NM_153212.2)	(NP_694944.1)				

ADNSHL, autosomal dominant nonsyndromic hearing loss.

### Known Causative Variants: *GJB2* p.R75Q and *MYO7A* p.T381M

**Figure 2:**
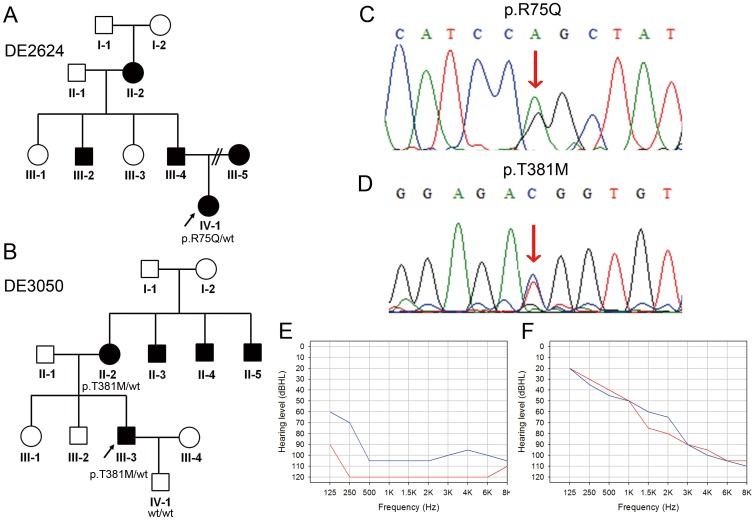
The *GJB2* p.R75Q mutation in Family DE2624 and the *MYO7A* p.T381M mutation in Family DE3050. (A) Pedigree and segregation pattern in Family DE2624. (B) Pedigree and segregation pattern in Family DE3050. (C) Sanger sequence of the *GJB2* p.R75Q mutation. Arrow: nucleotide change c.224G>A (p.R75Q) variant. (D) Sanger sequence of the *MYO7A* p.T381M mutation. Arrow: nucleotide change c.1142C>T (p.T381M) variant. (E) Audiogram of the proband of Family DE2624. (F) Audiogram of the proband of Family DE3050. Hearing levels of the right ear and left ear are marked with red and blue lines, respectively.

We identified 2 known causative variants, *GJB2* p.R75Q (c.224G>A) and *MYO7A* p.T381M (c.1142C>T), in Family DE2624 and Family DE3050, respectively (Figure 2). Variant reads of *GJB2* p.R75Q were 48% (120/251) of total reads in the proband of Family DE2624, suggesting heterozygosity of the allele (Table 2). p.R75Q has been associated to both autosomal dominant nonsyndromic hearing loss (ADNSHL) [26] and autosomal dominant SNHI with palmoplantar keratoderma [27]. The proband of Family DE2624 was a 5-year-old girl with profound hearing loss (Figure 2E) and normal temporal bone imaging results. Palmoplantar keratoderma was not observed in the affected members of Family DE2624, thus indicating that the phenotype associated with *GJB2* p.R75Q in this family was nonsyndromic hearing loss.

In the proband of Family DE3050, variant reads of *MYO7A* p.T381M were 56% (41/73) of the total reads, suggesting heterozygosity of the allele (Table 2). Heterozygosity for *MYO7A* p.T381M has been documented in 3 unrelated patients with nonsyndromic SNHI from Taiwan [28], and co-segregated with the phenotype of hearing impairment in the pedigree of Family DE3050 (Figure 2B). The proband of Family DE3050 was a 31-year-old man with progressive, moderate SNHI (Figure 2F) and no inner ear malformations (IEM) on temporal bone imaging studies. None of the affected members in Family DE3050 exhibited retinitis pigmentosa.

### Novel Causative Variants: *KCNQ4* p.S680F and *MYH9* p.E1256K

**Figure 3:**
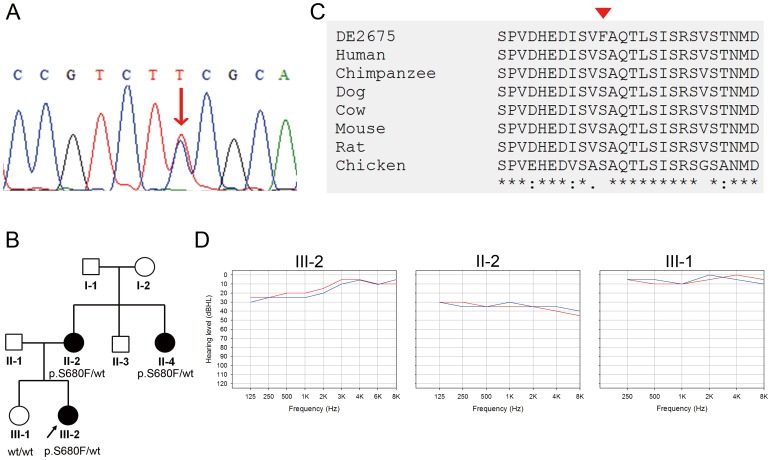
The *KCNQ4* p.S680F variant identified in Family DE2675. (A) Sanger sequence of the *KCNQ4* p.S680F variant. Arrow: nucleotide change c.1877C>T (p.S680F) variant. (B) Pedigree and segregation pattern in Family DE2675. (C) Evolutionary conservation of the p.S680 residue. Arrowhead: variant site. (D) Audiograms of the affected family members (II-2 & III-2) and an unaffected member (III-1) of Family DE2675. Hearing levels of the right ear and left ear are marked with red and blue lines, respectively.

In Family DE2675, we identified the p.S680F (c.1877C>T) variant in the *KCNQ4* gene, which has not been reported previously (Figure 3A). The pathogenicity of *KCNQ4* p.S680F was supported by its high SIFT (0.98) and PolyPhen2 (0.99) scores, as well as its absence in the 5400 NHLBI exomes, 1000 Genomes, and the 100 normal hearing controls of the present study (Table 2). Furthermore, heterozygosity for p.S680F co-segregated with the phenotype of hearing impairment in the pedigree (Figure 3B), and the amino acid residue p.S680 was evolutionarily conserved (Figure 3C). The proband (III-2) of Family DE2675 was a 7-year-old girl with mild low-tone hearing loss, and her mother’s (II-2) hearing loss began at adolescence and gradually deteriorated to mild SNHI until her current age (Figure 3D). Both exhibited normal temporal bone imaging results. By contrast, the elder sister (III-1) of the proband who did not segregate the p.S680F variant demonstrated a normal audiogram.

**Figure 4:**
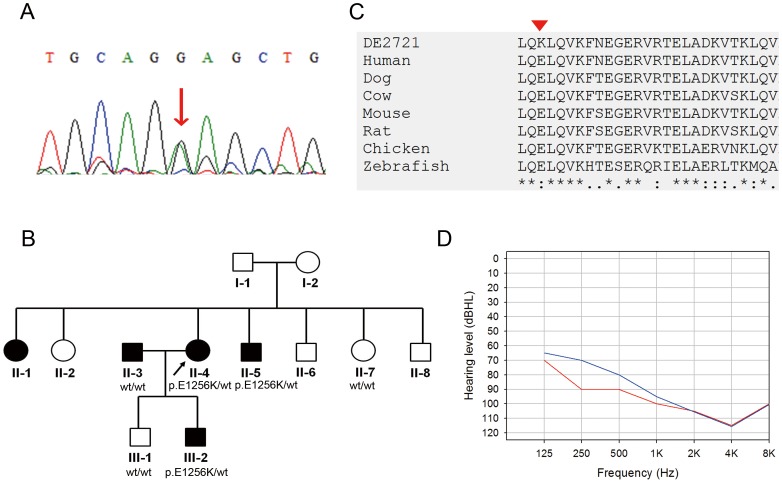
The *MYH9* p.E1256K variant identified in Family DE2721. (A) Sanger sequence of the *MYH9* p.E1256K variant. Arrow: nucleotide change c.3766G>A (p.E1256K) variant. (B) Pedigree and segregation pattern of Family DE2721. (C) Evolutionary conservation of the p.E1256 residue. Arrowhead: variant site. (D) Audiogram of the proband of Family DE2721. Hearing levels of the right ear and left ear are marked with red and blue lines, respectively.

In Family DE2721, we identified the p.E1256K (c.3766G>A) variant in the *MYH9* gene, which has not been reported previously (Figure 4A). The pathogenicity of *MYH9* p.E1256K was supported by its high SIFT (1.00) and PolyPhen2 (1.00) scores, as well as its absence in 5400 NHLBI exomes, 1000 Genomes, and the 100 normal hearing controls (Table 2). In addition, heterozygosity for p.E1256K co-segregated with the phenotype of hearing impairment in the pedigree (Figure 4B), and the amino acid residue p.E1256 was evolutionarily conserved (Figure 4C). The proband of Family DE2721 was a 57-year-old woman with profound SNHI that had progressed since 10 years of age (Figure 4D). One of her sons had segregated the *MYH9* p.E1256K variant and also exhibited progressive SNHI of moderate severity. The result of the temporal imaging study performed in the proband was normal. Comprehensive renal, hematological, and ophthalmological studies in the proband revealed normal results, and her affected son (III-2) also showed normal laboratory findings on complete blood count and serum renal profiles. Hematologic and renal disorders were ruled out in other affected members of Family DE2721 by history.

### Probable Non-causative Variant: *GJB4* p.C169W

**Figure 5:**
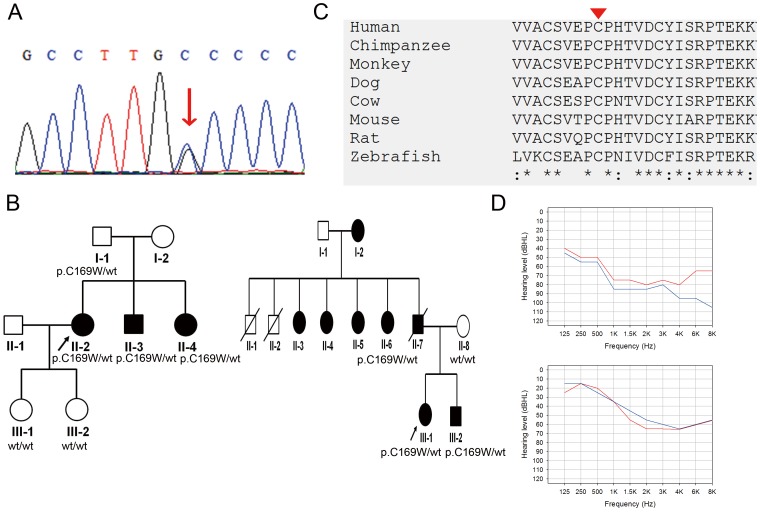
Probable non-causative *GJB4* p.C169W variant identified in Family DE2827 and Family DE3281. (A) Sanger sequence of the *GJB4* p.C169W variant. Arrow: nucleotide change c.507C>G (p.C169W) variant. (B) Pedigree and segregation patterns of Family DE2827 and Family DE3281. (C) Evolutionary conservation of the p.C169 residue. Arrowhead: variant site. (D) Audiograms of the probands of Family DE2827 (upper panel) and Family DE3281 (lower panel). Hearing levels of the right ear and left ear are marked with red and blue lines, respectively.

In Family DE2827 and Family DE3281, we identified the p.C169W (c.507C>G) variant in the *GJB4* gene (Figure 5A). In both Family DE2827 and Family DE3281, heterozygosity for *GJB4* p.C169W co-segregated with the hearing impairment phenotype in the pedigree (Figure 5B and 5C), except for case I-1 of Family DE2827, which exhibited normal hearing that corresponded to his age. The proband of Family DE2827 was a 44-year-old woman with progressive severe SNHI (Figure 5E, upper panel), whereas the proband of Family DE3281 was a 50-year-old woman with progressive SNHI of moderate severity (Figure 5E, lower panel). Both probands exhibited normal temporal bone imaging results. The SIFT and PolyPhen2 scores of *GJB4* p.C169W were 1.00 (Table 2). The amino acid residue p.C169 was evolutionarily conserved (Figure 5D), and heterozygosity for the nonsense mutation at this amino acid residue (i.e., p.C169X) has been related to nonsyndromic SNHI in a Han Chinese family from Taiwan [29]. However, the allele frequencies of *GJB4* p.C169W were as high as 2.8% and 3.0% in 5400 NHLBI exomes and 1000 Genomes, respectively. Among the 100 normal hearing controls, we also identified 6 carriers with *GJB4* p.C169W, revealing an allele frequency of 3.0% (6/200 chromosomes). Therefore, probably *GJB4* p.C169W is a non-pathogenic variant.

## Discussion

**Figure 6:**
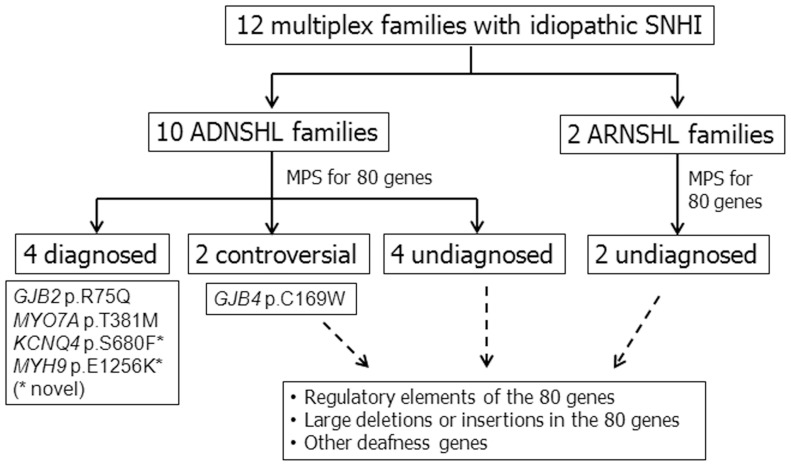
Summary of the genetic diagnostic results obtained in the present study. Twelve multiplex families with idiopathic SNHI, including 10 ADNSHL and 2 ARNSHL families, were subjected to MPS for 80 known human deafness genes. Four out of 10 ADNSHL families were diagnosed to segregate causative variants, including *GJB2* p.R75Q, *MYO7A* p.T381M, *KCNQ4* p.S680F, and *MYH9* p.E1256K. The *GJB4* p.C169W variant of controversial pathogenicity was identified in another two ADNSHL families. In addition to the 2 families with *GJB4* p.C169W, we were unable to achieve a genetic diagnosis in the other 4 ADNSHL families and the 2 ARNSHL families. The cause of SNHI in these undiagnosed families might be attributed to genetic defects in the regulatory elements of the 80 genes, large deletions or insertions of >100 bp in the 80 genes, or even other deafness genes. SNHI, sensorineural hearing impairment; ADNSHL, autosomal dominant nonsyndromic hearing loss; ARNSHL, autosomal recessive nonsyndromic hearing loss; MPS, massively parallel sequencing.

In the present study, we applied MPS to 12 multiplex families with idiopathic SNHI. We identified 5 variants in 5 different genes, which might have led to SNHI in 6 families compatible with autosomal dominant inheritance, including *GJB2* p.R75Q, *MYO7A* p.T381M, *KCNQ4* p.S680F, *MYH9* p.E1256K, and *GJB4* p.C169W (Figure 6). Four variants were classified as pathogenic variants, including 2 known variants (*GJB2* p.R75Q and *MYO7A* p.T381M) and 2 novel variants (*KCNQ4* p.S680F and *MYH9* p.E1256K). The remaining variant, *GJB4* p.C169W, was classified as a probable non-causative variant, as its allele frequency was as high as ∼3% in the general population.

Among the deafness-associated genes identified thus far, mutations in *GJB2* are the most common cause of monogenic hearing impairment worldwide. *GJB2* encodes a gap junction protein, connexin 26, and the major function of connexin 26 in the inner ear is to regulate potassium recycling [30]. To date, 2 mutations at the p.R75 amino acid residue of connexin 26 have been related to autosomal dominant SNHI with or without palmoplantar keratoderma: p.R75W [31], [32] and p.R75Q [26], [27]. *GJB2* p.R75Q was identified in 1 Turkish and 2 French families, with the severity of hearing loss ranging from mild to profound SNHI in the affected individuals [26], [27]. We failed to detect p.R75Q in our first pass of mutation screening using the SNaPshot multiplex assays, as p.R75Q was not included in the screening panels because it had never been detected in our previous genetic epidemiological survey [8].

Another known causative variant identified in the present study is *MYO7A* p.T381M. The human *MYO7A* gene consists of 48 coding exons, and encodes the unconventional myosin MYO7A with 2,215 amino acids [33]. Different *MYO7A* mutations might lead to a wide variety of phenotypes with different inheritance patterns, ranging from Usher syndrome type 1B [34], autosomal recessive nonsyndromic hearing loss (ARNSHL) (DFNB2) [33], [35], to ADNSHL (DFNA11) [36]. *MYO7A* p.T381M is located in the MYO7A motor head domain, and the previously reported 3 unrelated Han Chinese patients who segregated 1 allele of *MYO7A* p.T381M revealed nonsyndromic severe-to-profound SNHI [28]. Together with the results of this study, it is likely that *MYO7A* p.T381M is associated with ADNSHL.

In addition to the 2 known causative variants, we identified 2 novel variants, i.e., *KCNQ4* p.S680F and *MYH9* p.E1256K, in 2 families, respectively. The *KCNQ4* gene, which encodes the voltage-gated potassium channel KCNQ4 in outer hair cells, has been identified at the ADNSHL DFNA2 locus on the human chromosome 1p34 [37]. To date, 11 missense mutations, 1 nonsense mutation, and 2 deletion mutations of the *KCNQ4* gene have been documented in DFNA2 patients with various clinical phenotypes (The Deafness Variation Database: http://deafnessvariationdatabase.org) [38]. *KCNQ4* p.S680F is located at the cytoplasmic C terminus and contributes to progressive SNHI in the affected members of Family DE2675. The *MYH9* gene encodes myosin IIA heavy chain, which is involved in actomyosin-microtubule crosstalk, cell motility, and maintenance of cell shape [39]. Defects in *MYH9* have been associated with ADNSHL DFNA17 [40], as well as a plethora of autosomal dominant syndromes with hematologic and/or renal manifestations in addition to progressive SNHI [41]. As none of the affected members in Family DE2721 demonstrated symptoms other than SNHI, the p.E1256K variant might lead to nonsyndromic SNHI only.

The *GJB4* p.C169W variant was detected in 2 other autosomal dominant families. *GJB4* p.C169W fulfilled most criteria used for data filtering in the present study, including allele frequency <5% in the general population, high PolyPhen2 and SIFT scores, and evolutionary conservation of p.C169 amino acid residues. It is important to note, that the allele frequency of *GJB4* p.C169W is approximately 3% in the general population, which is, to some extent, higher than the convention definition of a mutation, i.e., the allele frequency of a mutation should be less than 1% [42]. It has been demonstrated that the allele frequencies of several common deafness mutations are higher than 1% in the general population, e.g., *GJB2* p.V37I (∼10% in some East Asian general populations) [43], [44], c.235delC (∼1.5% in the Chinese general population) [45], and c.35delG (∼1.3% in the European general population) [46]. However, all these mutations are recessive. As such, it is unlikely that *GJB4* p.C169W leads to ADNSHL given its high allele frequency in the population. However, there might be hypothetical possibility that this variant indeed contributes to SNHI through “autosomal dominant but low penetrance” or “oligogenic” inheritance pattern; we therefore still presented the results in this paper for the sake of completeness.

In addition to the 2 families with *GJB4* p.C169W, we were not able to obtain a genetic diagnosis in the other 4 ADNSHL families and the 2 ARNSHL families using the current MPS panel (Figure 6), although many affected members strongly suggest a genetic cause of deafness in these families. As the MPS diagnostic panel of the present study only targeted the coding sequences and splicing sites of known deafness genes, genetic defects in regulatory elements, e.g., un-translated regions (UTRs), enhancers, promoters, and intronic splicing regulatory elements, could not be examined. Large deletions or insertions of >100 bp, as well as inversions, might also be missed because of the short average read length of the Illumina platform used in our MPS diagnostic panel. Another possibility is that deafness in these families might be caused by mutations in genes other than the 80 known deafness genes targeted in the present study. For the latter possibility, whole exome sequencing might be helpful in identifying novel deafness genes in the future.

The MPS technology, via targeted sequencing of whole exomes [47] or specific chromosomal regions confined by linkage analysis and homozygosity mapping [48], has been proven to be a powerful tool for discovering novel deafness genes (for a detailed review, please refer to [49]). In addition to gene discovery, the diagnostic utility of MPS in addressing genetically heterogeneous nonsyndromic hearing loss has also been examined in several studies (Table 3). Shearer *et al*. performed targeted capture of 54 known deafness genes in 9 individuals, including 3 positive controls with mutations detected previously and 6 patients with idiopathic SNHI. Causative mutations were confirmed in all 3 positive controls, and genetic diagnosis was achieved in 5 of the 6 idiopathic SNHI patients [12]. Brownstein *et al*. developed a targeted capture pool composed of 246 deafness genes (85 human and 161 mouse genes), and applied it to 11 unrelated families with idiopathic SNHI, including 2 with ADNSHL and 9 with ARNSHL. They established genetic diagnosis in 2 ADNSHL and 4 ARNSHL families, and identified 4 novel mutations. Interestingly, a genetic diagnosis was made in all 5 Jewish families, but in only 1 of the 6 Palestinian families. A possible explanation for the difference in the diagnostic yield between populations, as proposed by the authors, is that inherited hearing loss in the Palestinian population is more heterogeneous than in the Israeli population because of historical marriage patterns [13]. Tang *et al*. selected 5 known deafness genes to test the diagnostic efficacy of MPS in 10 positive controls previously identified to have *GJB2* mutations by Sanger sequencing. They successfully confirmed the causative mutations in all positive controls and detected additional variants in the other selected genes [50]. De Keulenaer *et al*. screened 15 ARNSHL genes in 1 positive control and 3 ARNSHL families with MPS and identified a pathogenic mutation in 2 ARNSHL families of Iranian ancestry [51]. In the present study, we performed targeted capture of 80 known deafness genes in 10 ADNSHL and 2 ARNSHL families and identified causative variants in 4 ADNSHL families. In summary, MPS accurately detected causative mutations in the positive controls in these series and facilitated the genetic diagnosis in a certain proportion of idiopathic SNHI families. However, the diagnostic yield of MPS may depend on several factors, including the number of targeted genes, the MPS platform used, the inheritance pattern, and the ethnic background of the families tested.

**Table 3:** Summary of the 5 Series Investigating the Diagnostic Utility of MPS.

Reference	Targeted regions	Subjects enrolled for MPS	Main results
Shearer *et al.*	54 known deafness genes inhumans; exons and 50 bpflanking introns	3 positive controls with mutations detectedpreviously by Sanger sequencing and 6 patientswith idiopathic SNHI. The 6 idiopathic SNHIpatients included 4 withADNSHL and 2 with ARNSHL. [ethnicity: NA]	Causative mutations were confirmed in the 3 positive controls. Pathogenic mutations were identified in 5 of the 6 idiopathic SNHI patients, including 3 novel mutations. Genetic diagnosis remained elusive in an ADNSHL patient.
Brownstein *et al.*	246 genes responsible for deafnessin humans (85 genes) and mice(161 genes); exons and 40 bpflanking introns	11 unrelated families with idiopathic SNHI,including 2 with ADNSHL and 9 with ARNSHL.[ethnicity: 5 Israeli Jewish and 6Palestinian Arab]	Pathogenic mutations were identified in 2 ADNSHL and 4 ARNSHL families, including 4 novel mutations. Genetic diagnosis could be achieved in all 5 Jewish families, but in only 1 Palestinian family.
Tang *et al.*	5 selected known deafness genesin humans; exons and 50 bpflanking introns	10 positive controls previously detected tohave mutations in *GJB2* by Sangersequencing. [ethnicity: NA]	Causative *GJB2* mutations were confirmed in the positive controls, and additional variants in other selected targeted genes were identified.
De Keulenaer *et al.*	15 ARNSHL genes in humans;exons and most of the UTRs	1 positive control and 3 ARNSHL families(4 individuals).[ethnicity: the 3 ARNSHLfamilies included 2 Iranian and 1 Turkish]	The causative mutation was confirmed in the positive control. Pathogenic mutations were identified in 2 ARNSHL families, including 2 novel mutations.
This study	80 known deafness genes inhumans; exons and 100 bpflanking introns	12 multiplex families with idiopathic SNHI,including 10 with ADNSHL and 2 with ARNSHL.[ethnicity: all Han Chinese]	Causative variants were identified in 4 ADNSHL families, including 2 novel variants.

MPS, massively parallel sequencing; SNHI, sensorineural hearing impairment; ADNSHL, autosomal dominant nonsyndromic hearing loss; ARNSHL, autosomal recessive nonsyndromic hearing loss; NA, not available; UTR, untranslated region.

In conclusion, by applying the MPS diagnostic panel targeting 80 deafness genes to 12 multiplex Han Chinese families with idiopathic nonsyndromic SNHI, we identified 4 variants in 4 different genes, which might have led to SNHI in 4 families compatible with autosomal dominant inheritance, including *GJB2* p.R75Q, *MYO7A* p.T381M, *KCNQ4* p.S680F, and *MYH9* p.E1256K. Among them, *KCNQ4* p.S680F and *MYH9* p.E1256K are novel variants. Thus, MPS enables genetic diagnosis in multiplex families with idiopathic SNHI by detecting mutations in relatively uncommon deafness genes.

## Supporting Information

Figure S1 **The pedigrees of the 12 multiplex families recruited in the present study.** Families compatible with autosomal dominant inheritance and autosomal recessive inheritance are marked with AD and AR, respectively. Arrows indicate the probands.
(TIF)

Table S1 **The 80 known human deafness genes included in DA1.**
(DOC)
