# Supplementary figures and images for: Application of Massively Parallel Sequencing to Genetic Diagnosis in Multiplex Families with Idiopathic Sensorineural Hearing Impairment
Source: PLoS One. 2013 Feb 22;8(2):e57369. doi: 10.1371/journal.pone.0057369 (PMC3579845; doi:10.1371/journal.pone.0057369)

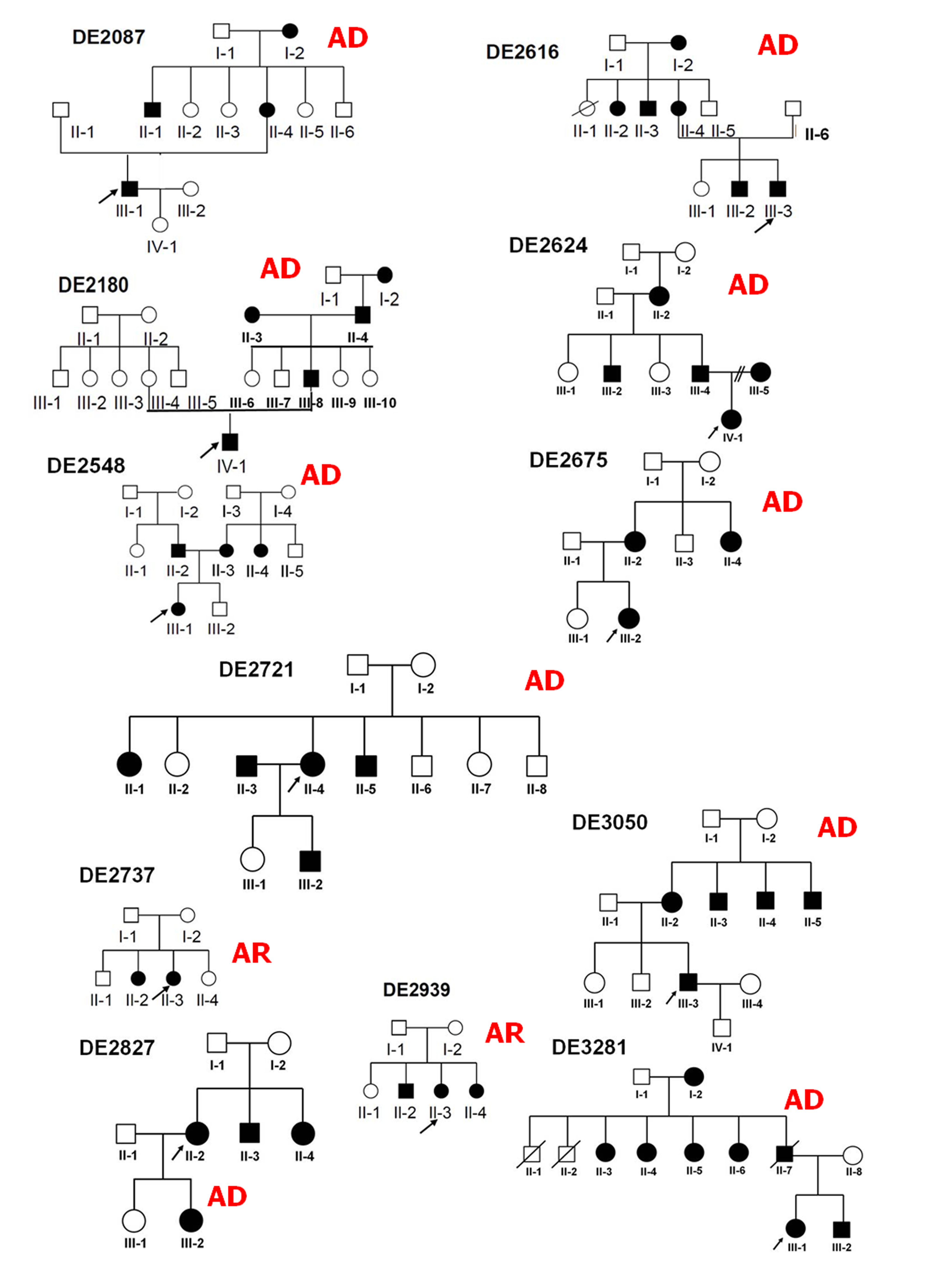

Supplement: Figure S1 — The pedigrees of the 12 multiplex families recruited in the present study. Families compatible with autosomal dominant inheritance and autosomal recessive inheritance are marked with AD and AR, respectively. Arrows indicate the probands. (TIF) [file pone.0057369.s001.tif]
