# Supplementary material for: Application of Massively Parallel Sequencing to Genetic Diagnosis in Multiplex Families with Idiopathic Sensorineural Hearing Impairment
Source: PLoS One. 2013 Feb 22;8(2):e57369. doi: 10.1371/journal.pone.0057369 (PMC3579845; doi:10.1371/journal.pone.0057369)
Supplement: Table S1 — The 80 known human deafness genes included in DA1. (DOC) [file pone.0057369.s002.doc]

**Supplementary table 1** The 80 known human deafness genes included in DA1

| *ESPN* | *DSPP* | *GRHL2* | *TMPRSS5* | *MYO1F* |
| --- | --- | --- | --- | --- |
| *GJB4* | *MARVELD2* | *MTAP* | *TECTA* | *ERCC2* |
| *GJB3* | *NR2F1* | *TMC1* | *MYO1A* | *MYH14* |
| *KCNQ4* | *DIAPH1* | *DFNB31* | *SLC17A8* | *SLC4A11* |
| *BSND* | *POU4F3* | *LHX3* | *GJB2* | *JAG1* |
| *KCNJ10* | *SPINK5* | *GATA3* | *GJB6* | *OTOR* |
| *OTOF* | *COL11A2* | *MYO3A* | *COCH* | *COL9A3* |
| *ATP6V1B1* | *LHFPL5* | *PCDH15* | *ESRRB* | *KCNE1* |
| *ERCC3* | *MYO6* | *CDH23* | *STRC* | *CLDN14* |
| *DFNB59* | *GJA1* | *PDZD7* | *CATSPER2* | *TMPRSS3* |
| *BCS1L* | *EYA4* | *USH1C* | *CRYM* | *MYH9* |
| *PAX3* | *TCF21* | *GSTP1* | *OTOA* | *TRIOBP* |
| *TMIE* | *ACTB* | *FGF3* | *MYO1C* | *TBL1X* |
| *SOX2* | *DFNA5* | *LRTOMT* | *PMP22* | *GJB1* |
| *CCDC50* | *SLC26A5* | *MYO7A* | *MYO15A* | *POU3F4* |
| *WFS1* | *SLC26A4* | *RDX* | *ACTG1* | *TIMM8A* |
